# The Effects of Alcohol and Drugs of Abuse on Maternal Nutritional Profile during Pregnancy

**DOI:** 10.3390/nu10081008

**Published:** 2018-08-02

**Authors:** Giorgia Sebastiani, Cristina Borrás-Novell, Miguel Alsina Casanova, Mireia Pascual Tutusaus, Silvia Ferrero Martínez, María Dolores Gómez Roig, Oscar García-Algar

**Affiliations:** 1 Neonatology Unit, Hospital Clinic-Maternitat, ICGON, BCNatal, C/Sabino Arana 1, 08028 Barcelona, Spain; CBORRASN@clinic.cat (C.B.-N.); MMALSINA@clinic.cat (M.A.C.); mireiapascualt@gmail.com (M.P.T.); OGARCIAA@clinic.cat (O.G.-A.); 2 Barcelona Center for Maternal Fetal and Neonatal Medicine, Hospital Sant Joan de Déu, 08028 Barcelona, Spain; sferrero@sjdhospitalbarcelona.org (S.F.M.); lgomezroig@sjdhospitalbarcelona.org (M.D.G.R.)

**Keywords:** alcohol, drugs of abuse, pregnancy, maternal nutrition, fetal nutrition, Fetal Alcohol Spectrum Disorder (FASD), Intrauterine Growth Restriction (IUGR)

## Abstract

The consumption of alcohol and drugs of abuse among pregnant women has experienced a significant increase in the last decades. Suitable maternal nutritional status is crucial to maintain the optimal environment for fetal development but if consumption of alcohol or drugs of abuse disrupt the intake of nutrients, the potential teratogenic effects of these substances increase. Despite evidence of the importance of nutrition in addicted pregnant women, there is a lack of information on the effects of alcohol and drugs of abuse on maternal nutritional status; so, the focus of this review was to provide an overview on the nutritional status of addicted mothers and fetuses. Alcohol and drugs consumption can interfere with the absorption of nutrients, impairing the quality and quantity of proper nutrient and energy intake, resulting in malnutrition especially of micronutrients (vitamins, omega–3, folic acid, zinc, choline, iron, copper, selenium). When maternal nutritional status is compromised by alcohol and drugs of abuse the supply of essential nutrients are not available for the fetus; this can result in fetal abnormalities like Intrauterine Growth Restriction (IUGR) or Fetal Alcohol Spectrum Disorder (FASD). It is critical to find a strategy to reduce fetal physical and neurological impairment as a result of prenatal alcohol and drugs of abuse exposure combined with poor maternal nutrition. Prenatal nutrition interventions and target therapy are required that may reverse the development of such abnormalities.

## 1. Introduction

The consumption of alcohol and drugs of abuse among pregnant women has experienced a significant increase in the last decades.

Prenatal alcohol exposure is considered one of the major public health challenges. The last published systematic review about the prevalence of Fetal Alcohol Syndrome (FAS), defined by growth retardation, facial malformations and central nervous system impairment, indicated that the five countries with the highest estimated prevalence of alcohol use during pregnancy were Ireland (60.4%), Belarus (46.6%), Denmark (45.8%), UK (41.3%), and Russia (36.5%). The average prevalence of alcohol use during pregnancy was the highest in the European region (25.2%) and the lowest in the Eastern Mediterranean Region (0.2%). In relation to the prevalence of FAS, the review indicates that the five countries with the highest prevalence of FAS are South Africa (58.5 per 1000), Croatia (11.5 per 1000), Ireland (8.9 per 1000), Italy (8. per 1000) and Belarus (6.9 per 1000). The global prevalence of FAS among the general population is estimated to be 1.4 per 1000 and in the general population of Europe about a quarter of women drink alcohol during pregnancy [1]. Previous research estimated that 4.3% of children born among heavy drinking pregnant women (defined as an average of two or more drinks per day, or five to six drinks per occasion) will have FAS, which is about three times greater than the quotient estimated among women in the general population who consumed any amount of alcohol during pregnancy [2]. FAS is a preventable disease but its prevalence could increase around the world in the next years. The average of alcohol use, binge drinking, and drinking during pregnancy are increasing among young women in a lot of countries; moreover, a lot of pregnancies in developing and developed countries are unplanned, with the increased risk of involuntary exposition of the embryo to alcohol in the earliest stage of pregnancy, when brain development is more sensitive to its effects [3,4].

In Spain the prevalence of alcohol consumption during pregnancy through maternal hair analysis at the delivery is about 60%. Although the media and government continue informing about the negative effects of alcohol consumption during pregnancy, pregnant women continue to consume alcohol frequently (defined as more than seven drinks/week) or binge drink (when more than five drinks are consumed per occasion) [5].

Recent estimates of the prevalence of cannabis use among pregnant women in the US range between 3% and 16%. Population-based surveillance data from the National Survey on Drug Use and Health concludes that cannabis use among pregnant women in the US has increased as much as 62% between 2002 and 2014. The abuse of cannabis has increased six- to seven-fold since the 1970s. There is an increasing trend in prenatal cannabis use, due to the conception of need of cannabis for medical use, cannabis harmlessness, and increased access to this drug [6]. Data from 2007–2012 National Surveys on Drug Use and Health, a cross-sectional nationally representative survey, identified pregnant and nonpregnant women 18–44 years of age and they found that 10.9% of pregnant and 14.0% of nonpregnant US women of reproductive age used marijuana in the past year during 2007–2012, among whom 3.9% of pregnant and 7.6% of nonpregnant women used it in the past month. Moreover, 18.1% of pregnant and 11.4% of nonpregnant women met criteria for abuse and/or dependence [7]. In France, 1.2% of women reported having used cannabis during pregnancy [8].

A recent self-reporting survey by The National Survey on Drug Use and Health from 2002–2003 estimated that 4.3% of pregnant women aged 15 to 44 years reported illicit drug use. Approximately 250,000 women in the United States, of whom 90% are of childbearing age, meet criteria for intravenous drug abuse. This suggests that approximately 225,000 infants born each year could be exposed to illicit drugs in the prenatal or postpartum time period [9,10].

Cocaine is a stimulant drug most often abused in the intranasal form, but can also be injected or smoked (commonly known as crack cocaine). Crack is most often used in Latin countries, Brazil being the highest world consumer, where the estimated prevalence of use is over 0.81%. Population studies in United States suggested that cocaine abuse occurs in 1.1% of young people. However, hair and meconium screening for cocaine in American newborns suggested that rates of prenatal cocaine exposure may be nearly 10-fold higher. Similar results have been obtained in newborn screening of the Canadian and European population [10,11,12]. It has been described by the Alcohol Drug and Pregnancy Team (ADAPT) at National Women’s Hospital of New Zealand, that there has been a dramatic increase of women who have used Methamphetamine (MA) drug during their current pregnancy. In 2001, 10% (6/60) of the total ADAPT referrals were due to methamphetamine use and associated problems. This escalated to 59% (34/58) in 2003 [13].

Opioid use during pregnancy has also increased drastically in recent years. The percentage of Medicaid-enrolled women who filled at least one opioid prescription during pregnancy increased 23% during 2000–2010, from 18.5% to 22.8% [14]. The prevalence of opioid abuse or dependence among pregnant women has increased from 1.7 per 1000 delivery admissions in 1998 to 3.9 in 2011 [15]. The 2010 South African Community Epidemiology Network on Drug Use (SACENDU) statistics revealed that 5–20% of patients in specialist treatment centres in South Africa (SA) use heroin as their primary illicit drug of choice. In SA, heroin is principally used by individuals aged 22–30 years, and 20–40% of those treated for heroin abuse are female, which is a higher percentage than for most other illicit substances [16].

Proper nutritional status is critical in producing healthy offspring. When maternal nutritional status is damaged due to the toxic effects of alcohol or drugs of abuse, there is a lack of essential nutrients required for the correct growth of developing fetus that results in suboptimal health outcomes [17]. The recent literature has focused on maternal nutritional status and alcohol abuse in pregnancy but recent information about nutritional status of drug abusers during pregnancy is scarce. In addition, drug consumers generally abuse of multiples substances and have behavioural risk factors that undermine nutritional status such as needle-sharing, unprotected sex, and sex with multiple partners, leading them to the highest risk of human immunodeficiency virus (HIV) infection; so the altered nutrition of the mother is multifactorial and not only depends on specific substance [18].

The aim of this narrative review was to analyse the existing studies regarding the effects of drugs of abuse and alcohol on maternal nutritional status and fetal nutrition during pregnancy. A search for English written articles was performed using MEDLINE/PubMed databases (from 1970 up to 15 June 2018). The search was based on the following keywords: alcohol, nutrition, pregnancy, addiction, cocaine, methamphetamine, cannabis or marijuana, heroine or opioid, and fetus. Studied were indentified and examined for methodology and key results. Researches about preconception period were also analyzed. We included World Health Organization (WHO) guidelines for alcohol and drugs of abuse in general population and in pregnancy; moreover, we included scientific papers about the nutritional status of mothers, reflecting fetal nutrition in order to emphasize the detrimental nutritional effects of alcohol and drugs of abuse in pregnancy (Table S1). Especially, our aim was to highlight the effect of these substances on micronutrients to find a target therapy that could avoid fetal complication.

We did not analyse the effect of drugs of abuse and alcohol on pregnancy or on fetal development, neither the effect of nutritional status of the mother on drugs of abuse and alcohol.

## 2. Review

### 2.1. International Guidelines on Alcohol in Pregnancy

Alcohol is a psychoactive substance with dependence-producing properties. WHO alcohol guidelines exposed that alcohol-related harm is determined by the volume of alcohol consumed, the pattern of drinking, and, on rare occasions, the quality of alcohol consumed. Heavy episodic drinking (HED) is defined as consumption of 60 or more grams of pure alcohol (>6 standard drinks in most countries) on at least one single occasion at least monthly. Harmful use of alcohol is defined as a pattern of alcohol use that is causing damage to physical or mental health. Alcohol dependence typically include a strong desire to consume alcohol, difficulties in controlling its use, persisting in its use despite harmful consequences, increased tolerance, and sometimes a physiological withdrawal state [19]. Pattern of Drinking Score (PDS) reflect how people drink and are based on the following drinking attributes: the usual quantity of alcohol consumed per occasion; festive drinking; proportion of drinking events when drinkers get drunk; proportion of drinkers who drink daily or nearly daily; drinking with meals; drinking in public places [20].

The frequency of alcohol abuse, consumption pattern, and period of exposure during fetal development are critical factors linked with the severity of the distinct Fetal Alcohol Spectrum Disorder (FASD) features. Chronic, daily heavy alcohol use or frequent intermittent alcohol use is much more toxic to the fetus than acute, moderate alcohol consumption. It has been studied that a major risk to the fetus exists with the chronic consumption of >6 drinks/day. Consuming high, frequent amounts of alcohol during a single drinking episode, known as “binge drinking”, also increases blood alcohol concentrations and could be teratogenic [21].

International Guidelines of alcohol consumption during pregnancy are based on the assessment of the evidence concerning potential harms of alcohol for the developing fetus.

According to recent guidelines of Autralia [22], Canada [23], Denmark [24], France [25] and USA [26], although the risk of birth defects is greatest with high, frequent maternal alcohol intake during the first trimester, alcohol exposure throughout pregnancy (including before pregnancy) can have detrimental consequences for development of the fetal brain. It is not clear how the effects of alcohol are related to the dose and if there is a setpoint above which adverse effects occur. Heavy drinking is the greatest risk and the timing of exposure is important but not all ‘heavy’ drinkers will have an affected child. After fertilization, most of the organs grow very quickly during the embryonic period in the early weeks of pregnancy, and the organs continuously develop until birth. The teratogenic effects of alcohol are more toxic during embryogenesis; development can be disrupted especially during the first trimester [17].

However, several policies underline that a safe level has not been established and conclude that not drinking is the safest option. WHO guidelines recomend absteinance of drugs during pregnancy and programs of detoxification if the pregnant woman is addicted [27].

### 2.2. Effects of Alcohol Exposure on Maternal Nutritional Status

Nutrition and alcohol toxicity are relationed and this interaction may potentially increment or protect against alcohol’s teratogenicity. Alcohol consumption can adversely affect the quality and quantity of proper nutrient supply and energy intake also during the preconception period. Diet quality is poorest among the drinkers who consumed the high quantity with lower frequency and best among the drinkers who consumed low quantity but with higher frequency [28]. When consumed in excess, alcohol interferes with the nutritional status of the drinker, moreover many alcoholics do not consume a balanced diet. Light to moderate drinkers who consume one to two glasses or less of an alcoholic beverage per day, consider those beverage a part of their normal diet and acquire a certain number of calories from alcohol in substitution of calories from other nutrients. Excessive alcohol abuse may impair the absorption into the body and the utilization of various nutrients. Accordingly many alcoholics, heavy drinkers particularly can suffer from various degrees of malnutrition. Primary malnutrition occurs when alcohol replaces other nutrients in the diet, resulting in overall reduced nutrients intake. Secondary malnutrition occurs when the drinker consumes adequate nutrients but alcohol interferes with the absorption of those nutrients from the intestine so they are not available to the body. Alcohol can interfere with the uptake of essential aminoacids and vitamins, particularly B_1_ (thiamine), B_2_ (riboflavin), B_6_ (pyridoxine), vitamin A and C and folic acid. The severity of these deficiencies correlates with the amount of alcohol and with the corresponding decrease in vitamin intake. Many drinkers who consume more than 30% of their total calories as alcohol ingest less than the recommended daily amounts of carbohydrates, proteins, fats, vitamins and minerals, such as calcium and iron. Moreover, malnourished alcoholics break down the alcohol more slowly and therefore develop higher blood alcohol levels that aggravate this condition [29].

There is increasing evidence that poor maternal nutrition can compromise the healthy fetus development moreover it is known that women who drink alcohol before or during pregnancy often have poor nutritional status, especially heavy drinkers. May et al., studied risk factors for FAS among three distinct samples of women before, during and after pregnancy in Plains Indian Women and Women from South Africa. The study included big samples and found that the major risk factor of delivering children with FAS was higher maternal age at time of pregnancy. Other variables of maternal risk for FAS were binge drinking, heavy intergenerational drinking in the mother’s extended family, and a longer length of drinking career of the mother. A woman’s consumption of alcohol in the months just before pregnancy was key to the risk for FAS. Body mass index (BMI), lifelong and current nutrition had a substantial impact on relative risk for FAS: The mother of children with FAS had low BMI and small women are more likely to have a lower average of drinking for producing FAS symptoms than larger women [30]. Malnutrition during pregnancy and genetic susceptibility to alcohol toxicity result in a variety of brain dysfunctions, and it is responsible for various developmental failures and neurocognitive disease. Alcohol exerts its neurotoxic effects on the developing brain directly by acting on fetal brain tissues, and indirectly by interfering with placental physiology or by impairing the mother’s physiology and nutrition. Alcohol abuse in pregnancy is frequently associated with poor nutrition that can potentially increase brain damage [31].

Pregnant women commonly do not consume recommended amounts of micronutrients, including several that may be especially important in FASD, such as choline, folate, vitamin B_12_, iron, and vitamin A [32]. Alcohol can aggravate this micronutrient deficit. A study of non-pregnant alcohol abusers in the United Kingdom found that all participants had low intake of vitamin E and folate, and most had low intake of other nutrients like selenium, calcium, zinc, Vitamins A, B_1_, B_2_, B_6_, C and D [33].

May et al., recruited two population case-control studies in rural areas of the Western Cape province of South Africa and reported the diets of mothers with FASD children compared to mothers of normal control children who attended the same school, using a 24-h recall for dietary record. In the first study, mothers of children with FASD showed poor maternal nutritional status, characterized by low BMI and lower intake of vitamins A, C, D, E, B_2_, calcium, omega-3 fatty acids and choline if compared to mothers of control children [34]. In the second, May et al., recorded the intake of vitamin D, C, thiamin, pantothenic acid, B_12_, phosphorus, magnesium, selenium, sodium, potassium, Eicosapentanoic acid (EPA), decosapentanoic acid (DPA) and docosahexaenoic acid (DHA); however, all mothers had insufficient intake of micronutrients and mothers of children with FASD did not report lower intake for any nutrient. On the contrary, they showed that mothers of children with FASD consumed more total protein, vitamin E, C, B_6_, magnesium, phosphorus, EPA, DHA and DPA than mothers of controls but these differences did not have biological significance since all mothers were found to be deficient because excessive alcohol consumption nullified the beneficial effects of additional nutrient intake. Despite an increase in total food consumption in mothers of children with FASD, there was no corresponding increase in diet quality [35]. There are some limitations of this data: dietary maternal intake was not recorded during pregnancy, but was recollected by a single 24-h questionnaire 7 years after delivery.

Carter et al., studied longitudinally heavy drinking Cape Coloured pregnant women compared to nondrinking controls, recording anthropometry by arm skinfolds and dietary intakes of energy, protein, fat and 25 major micronutrients from three 24-h recall interviews. They showed that all women of both goups gained poor weight during pregnancy. Neither Alcohol consumption was related to triceps or biceps skinfolds nor to energy intake or intake of carbohidrates, protein, or fat. More than 85% of the interviewed women (both alcohol-consuming and not-consuming) reported inadequate intake of nutrients like fiber, calcium, copper, iodine, iron, zinc, choline, folate, vitamin C and D and more than 50% had inadequate intake for magnesium, selenium and thiamin. Alcohol was weakly associated to higher phosphorus, choline and vitamin B12 intake. Drinking frecuency was associated with a lower intake of vitamin C and occasional drinking was associated with more intake of vitamin D. This data concluded that the teratogenic effects of alcohol are not related to poor nutrition of heavy drinking mothers, but the study had the limitation of 24-h recall interviews and was limited to a specific population [36]. A prospective cohort data conducted in moderate to heavy drinking pregnant women from Western Ukraine showed that blood choline levels were similar to those of non-drinking pregnant women. Multivitamin supplementation was associated with a better score of the Bayley Scales of Infant Development in children at 6 months. One limitation of this data was the problematic population of Ukraine that is high-risk drinkers with more social and emotional problems [37].

To our knowledge, the majority of the few data published about the negative effect of alcohol on nutritional status of mothers, showed a lack of average intake of micronutrients, particularly vitamins, in heavy drinking pregnant or non-pregnant women, suggesting that the toxic effects of alcohol are related to the decreased levels of antioxidants and increased levels of toxic metabolites including acetaldehyde [38]. For example, the depletion of maternal vitamin A can alter neurological development in the fetus because alcohol competes with retinol in the metabolic pathway involving the enzyme alcohol dehydrogenase (ADH) [39]. A deficiency of folate is caused by low intestinal absorption in acute and chronic alcoholics, and is also attributable to decreased hepatic uptake and increased urinary excretion [40]. Choline is the principal micronutrient related to brain development and cognitive function making part of the cell membrane (phospholipids, phosphatidylcholine) and low levels of choline are included in the molecular etiology of FASD especially neurodevelopmental abnormalities [41].

A recent study analysed plasma levels of zinc (Zn) and Copper (Cu) in heavy drinking pregnant women in Ukraine and Russia, showing that mothers who consumed alcohol during pregnancy had lower levels of these minerals than non-drinking mothers [42]. Alcohol decreases serum levels of zinc because it causes a low intake of intestinal absorption and increases urinary excretion. Ethanol increases maternal liver metallothionein that sequesters zinc, resulting in a reduction in plasma zinc [43] with abnormal consequences on brain growth and function.

In animal models, selenium deposits and plasma concentrations are low in chronic alcoholics because of malabsortion and increased production of free radicals resulting from alcohol pathways [44]. In addition, pregnant alcohol abusers have decreased maternal intake of n–3 Fatty Acids-rich foods, so maternal DHA status and the placental transfer of DHA to the fetus also decreased [45].

Ethanol exposure has also been shown to alter Iron (Fe) regulation and homeostasis, although reported data did not find an association of iron deficiency and drinking pattern during pregnancy. Only a small group of very heavy drinkers (eight or more drinks per day) pregnant women had higher iron depletion without anemia [46].

### 2.3. Effects of Drugs of Abuse Exposure on Maternal Nutritional Status

The most common illicit drugs consumed during pregnancy are cocaine, methamphetamine (MA) and marijuana. In general, use of illicit drugs produces multiple nutrient deficiencies and malnutrition because they alter intake behaviors and taste, leading to severe weight loss. Poor nutritional status could impair immunity and it is the most common cause of immunodeficiency [47]. The use of these drugs suppresses appetite and affects food habits leading drug addicts to crave ‘empty-energy’, potentially nutrient-deficient foods that cause micronutrient deficiency [48].

The majority of drug addicts present below-normal BMI, biochemical values, and clinical signs of nutrient deficiency because of the consumption of poor quality nutrient-deficient foods [49]. The clinical signs of nutrient deficiency, particularly, are associated with micronutrient deficiencies [48]. The decreased BMI, haemoglobin and protein levels in the drug addicts respectively indicate the presence of chronic energy deficiency, anemia and protein–energy malnutrition. In addition, deficiency of antioxidant vitamins (A, C, E) in this population has recently been reported. As nutritional status affects the immune system, the majority of drug addicts (about 60%) had sexually-transmitted diseases. This outcome is consistent with the synergistic relationship between malnutrition and infections like HIV [50].

#### 2.3.1. Methamphetamine (MA)

MA are highly addictive powerful Central Nervous System (CNS) stimulants with a profound ability to increase wakefulness and focus. The principle mechanism of action is increased release of norepinephrine, serotonin, and dopamine from neurons within the brain. At the same time, amphetamines inhibit re-uptake of these neurotransmitters.

Despite adverse effects on fetal circulation by inhibiting these neurotransmitter transporters, MA use, particularly among young pregnant patients, appears to be increasing. Researches on MA use in the preconception period and pregnancy are conducted with difficulties because users of MA will commonly use other illicit drugs while pregnant, making it hard to separate the effects of MA from those of other illicit drugs. As with virtually all other drugs of abuse, there is an important confounding effect. More important, perhaps, is the association of MA use with risky sexual behaviors, teenage pregnancy, and potential increased risk of sexually transmitted infections according to desregulated lifestyle [51].

Abuse of MA has been associated with poor maternal weight gain and nutritional status. Generally, addicted women consume multiple substances and most of them have fairly powerful appetite suppressant properties. Moreover, the psychosocial risk factor as poverty and high incidence of psychopathology aggravate the undernutrition. Management of poor maternal nutrition is relatively hard because sometimes little can be done to change established maternal behaviors in regard to nutrition. There is a significant number of substance-abusing pregnant patients who may be severely underweight; both of these conditions suggest poor overall nutritional status, if overeating is present it is often a consequence of abstaining from MA addiction [52].

Carter et al., found that pregnant women addicted to MA had smaller biceps skinfold thickness, a measure of body fat, and they had significant lower BMI if compared to non-addicted pregnant women. Moreover, they showed lower intake of vitamin C and carbohydrates [35]. In experimental animals studies, MA has been shown to have vasoconstrictive effects resulting in decreased uteroplacental blood flow and fetal hypoxia, and anorexic effects on the mother which may result in intrauterine growth retardation (IUGR) [53].

#### 2.3.2. Cocaine

Human studies have shown that malnutrition in a cocaine-addicted population may be multifactorial and could involve lower caloric intake, abnormal metabolic and gastrointestinal functions, and other deleterious health drug effects. Cocaine acts as an appetite suppressant that tends to reduce body weight due to its anorexigenic effect [54]. A cocaine-addicted population of Brazil was found to have hemoglobin and hematocrit levels below normal, associated with a poor diet in micronutrients, including iron, as well as macronutrients especially an insufficient protein intake. They were also found to have a dysregulation of lipid metabolism characterized by low levels of HDL cholesterol and high levels of triglycerides, LDL cholesterol, total cholesterol, and also presented glucose alteration, but at lower percentages [55]. Islam et al., found lower concentrations of antioxidant vitamins E, C, and A in this population, suggesting a lack of access to certain foods [47]. However t data about the effects of cocaine use on maternal nutritional status are scarce. In one study, pregnant women with urine assays positive for cocaine weighed significantly less before the pregnancy, had lower hematocrit levels at the time of prenatal registration, and gained slightly less weight during the gestation than did those with negative assays [56]. Pregnant women who used cocaine or cannabis had low levels of ferritine and folate, these were particularly decreased in those women with higher drug concentrations. Increased leukocyte counts were also related with serum concentrations of cocaine and other drugs, which suggests that drug-related inflammation may partially explain the maternal weight effect [57]. Prenatal cocaine exposure has been associated with a variety of adverse effects in humans, including poor maternal weight gain, spontaneous abortion, placental abruption, premature and precipitous labor, fetal distress, IUGR, various birth defects, and neonatal neurobehavioral deficits [58]. Fetuses of cocaine-addicted pregnant women showed low birthweight and low cranial circumference, although the deficit in birth weight did not achieve significance when pre-pregnancy weight and maternal weight gain were controlled for in the analysis; significant decreases in birth length and head circumference remained [59]. Thus, these data suggest that the association between cocaine use and growth retardation may be partially but not completely mediated by nutritional factors. Other factors, such as cigarette smoking, alcohol consumption, and other drug abuse, which were not controlled for in all the studies, may also have confounded some of the reported adverse effects. Isolating the influence of cocaine from other factors would nevertheless be difficult, since cocaine use is often accompanied by abuse of other substances as well as other life-style patterns that may be detrimental to the fetus.

Animal studies, on the other hand, can control for many confounding variables, providing a clearer view of drug-induced effects. All cocaine doses in animal models resulted in significant decreases in maternal weight gain, less food intake and a delay in weight recovery after delivery; the severity of these effects correlated with the daily dosage. Studies on sprague rats have properly demonstrated that undernutrition is a sufficient cause of fetal weight reduction at doses of 50 mg/kg/day [58]. Lethal effects in pregnant women and their fetuses were most often presented at 60 mg/kg/day of cocaine [60].

In animals models, cocaine increased water consumption during pregnancy, which suggests that cocaine, like other drugs, causes excess thirst. This effect could be caused by increased locomotor activity or dryness in the mouth similar to that caused by adrenaline and other sympathometic drugs or by a possible diuretic effect. Cocaine provoked diarrhoea in some cocaine-treated rats. This suggests that cocaine can act as a gastrointestinal irritant, causing malabsorption and fluid loss in at least some animals receiving very high doses. Cocaine-induced diarrhoea or diuresis could cause natriuresis (sodium loss) and the loss of other electrolytes and nutrients causing malnutrition [57].

#### 2.3.3. Cannabis

Few data report the nutritional status of pregnant marijuana users, nor is it known what effect marijuana exposure may have on specific nutrients. Even if in an experimental study performed by Abel in 1971 marijuana smokers consumed significantly more food [61], studies on women who have consumed marijuana during pregnancy have provided conflicting results regarding their nutritional status. Some studies support that marijuana users gained more weight during pregnancy, as described by O’Connell CM and Fried PA, who found that addicted women consumed significantly more calories and protein [62]; or Van Gelder MM et al., who reported that cannabis users were more likely than nonusers to have excessive weight gain during pregnancy [63]. Conversely, another study reported that women who had a positive assay for marijuana use weighed slightly less before pregnancy and gained significantly less weight during gestational period as compared with those who had a negative assay. These authors found as well that the infants whose mothers had positive urine assays for marijuana or cocaine had decreased birth somatometry [59].

Van Gelder et al., also described that women who reported use of any illicit drug, being aware of the limitation that it is likely underestimated, because respondents often falsely deny their consumption, were less likely to have used folic acid in the periconceptional period and during pregnancy, and they were also more often underweight (BMI < 18.5 kg/m^2^) than women who did not report use of illicit drugs [63]. Knight et al., after screening for blood concentrations of some illicit drugs and serum folate, vitamin B12, ferritin and ascorbic acid, showed that subjects whose serum values were above the ADAMHA/NIDA (National Institute on Drug Abuse) ranges for marijuana, phencyclidine and cocaine, had less concentrations of folate and ferritin than those of subjects with lower serum drug levels [57]. Roberson et al., used the Pregnancy Risk Assessment Monitoring System data of Hawaii and found that women who used marijuana in pregnancy, although its anti-emetic legal prescription, were more likely to report severe nausea, that could be explained by rare cannabinoid hyperemesis syndrome which would justify undernutrition in this population [64].

In a study realized in animals models, female wistar rats were exposed to cannabis smoke, placebo smoke, or no smoke while concurrently consuming one of three diets differing in protein concentration (8%, 24%, 64%). Twelve variables were affected by the low-protein diet; eight were significantly potentiated when undernutrition was combined with cannabis treatment, these included a lengthened gestation period, an increase in occurrence of stillbirths and litter destruction, and decreased activity in the rat pups; so, low-protein diet potentiated worse effect of cannabis [65].

#### 2.3.4. Heroine

Heroin is the most common illicit opiate consumed in pregnant women. Generally, women who use heroin consume other harmful substances, such as tobacco, alcohol and cocaine, all of which have their own potential toxic effects on pregnancy. Therefore, it is difficult to separate the effects of heroin from these other substances. In addition, intravenous drug use is a risk factor for many infectious diseases, including HIV infection [52]. Opiates are CNS depressants and analgesics, and they create physical and psychological dependence. Opioid misuse is defined as use of heroin or non-medical use of prescription-type pain relievers in the past month.

Making comparisons between pregnant and nonpregnant women of varying age groups provides an opportunity to determine whether opioid misuse differs by pregnancy status in the same way across different age groups. Nonpregnant women aged 18 to 25 (3.9%) and nonpregnant women aged 15 to 17 (3.6%) were more likely to misuse opioids in the past month than nonpregnant women aged 26 to 34 (2.3%) and nonpregnant women aged 35 to 44 (1.6%). Similarly, among pregnant women, those aged 15 to 17 and 18 to 25 were more likely to misuse opioids in the past month than pregnant women aged 26 to 34 (2.8 and 1.5% vs. 0.5%) [66].

Studies about opiate addiction disorders showed extreme nutritional deficiencies of key proteins, fats, vitamins, minerals like zinc, iron, calcium, chromium, magnesium, potassium and other essential nutrients. Drug abusers have severe weight loss and dietary patterns changes, which impair their ability to digest carbohydrates and other nutrients efficiently [67]. Metabolic problems are often associated with heroin, cocaine, and ecstasy drug although they produce a variety of medical problems. The use of heroin has been implicated in blood sugar disorders with different mechanisms. Fasting insulin levels were found to be four times higher in heroin addicts than in control subjects and insulin resistance caused by opioid use may be coupled with beta cell dysfunction [68].

In opiate addicts unhealthy eating behaviours have been shown due to lack of nutritional knowledge, food preparation manners, and environments [69,70].

During withdrawal from heroin, nicotine, marijuana, and cocaine, addicted people gain or lose weight, this is caused by major changes in food intake selection. Nutrition is related with conditions and diseases, such as diabetes, which decrease sensitivity to dependence on morphine and vitamin D deficiency, that decrease morphine dependency as well as protein deprivation which generates preferential fat intake with low cocaine use [48].

Heroin addicts consume less than the minimum daily quantity of vegetables, fruits and grains recommended by the food pyramid and eat more portions of sweets [69].

The carbohydrate-metabolism health problems could be reversed by increasing the dietary intake of protein and reducing simple carbohydrates in the form of vegetables and whole grains [71]. Management of pregnant substance abusers must address the needs of poorly nourished, homeless, and /or incarcerated pregnant substance abusers. In addition to education about nutrition and weight gain, some of these women may need referral to food assistance programs and shelters, and provision of transportation vouchers and prenatal multivitamins [72].

Outreach and educational resources targeting younger pregnant women and women living below the federal poverty level about the dangers of misusing prescription pain relievers may be especially beneficial [66]. No specific studies about nutritional impairment in pregnant women addicted to heroin have been reported. There is no specific data about definition and type of patterns of drug use, abuse, physical dependence, psychological dependence or addiction.

For opioid dependence, in addition to recommending cessation of opioid use, that is difficult for the environment of the mother previously described, there is the option of prescribing long-acting opioids such as methadone and buprenorphine to maintain stable opioid levels Although this treatment approach includes a risk of neonatal opioid withdrawal symptoms, opioids are essentially non-toxic at stable levels. Methadone maintenance treatment, itself, is not a favorable approach until it is coupled with proper diet due to the negative role of vitamin and mineral deficiencies in the withdrawal process. Using methadone among pregnant women with opioid use disorder improves outcomes for mothers and infants compared with the outcomes for mothers and infants who receive no treatment. Maintenance therapy with methadone or buprenorphine provides a steady concentration of opioids in the pregnant woman’s blood, preventing the fetus from repeatedly experiencing cycles of opioid toxicity and withdrawal.

### 2.4. Effects of Alcohol and Drugs of Abuse Exposure on Fetal Nutritional Status

Proper maternal nutritional status is crucial to maintain the optimal environment for fetal development. All gestational periods are critical and need perfect balance for the interchange of nutrients across the placenta and consequent correct fetal growth. Nutrients available to the fetus are dependent on the mother’s metabolism, her partitioning of nutrients and the placental transport mechanism. If maternal nutritional status is compromised, essential nutrients are not available to the fetus. Lighter (lower BMI) women population who binge drink are less able to eliminate alcohol allowing more alcohol to cross the placenta. Conversely, in mothers with high BMI the additional adipose tissue helps distribute the alcohol, and therefore, protects the fetus. Ethanol crosses the placental barrier so maternal consumption generates prolonged periods of exposure in the fetus causing severe damage to developing structures [73]. If fetal exposure to alcohol increases for mother malnutrition, the consequence is the increment of the risk of teratogenic effects resulting in suboptimal outcomes like IUGR or FASD. High blood alcohol concentrations often decrease the transfer across the placenta of essential nutrients required for fetal development [17]. After alcohol consumption, placenta generates oxidative stress (ROS) and changes in blood flow impairing maternal circulation to placenta [74]. In addition, ethanol decreases nitric oxide (NO) availability causing an imbalance of fetal blood flow, decreasing the delivery of nutrients to the fetus and generating IUGR and FASD [73,74].

Alcohol can alter the fetal supply of nutrients through multiple mechanisms including the decrease of intake micronutrients, malabsorption and increased urine excretion of key micronutrients and altered placental transportation [75,76]. Data suggest that mother of children with FASD have suboptimal levels of nutrients not severe deficiency, but this is sufficient to act as a factor for developing FASD so the low BMI of the mother can exacerbate the toxic effect of alcohol [34].

Cocaine and heroin cause vasoconstriction that leads to fetal hypoxia [77] and reduced placental transport of nutrients to the fetus. Since cocaine, like MA, acts as a suppressant of appetite [78,79], an inadequate maternal diet may play a role in the growth retardation seen in fetuses of cocaine abusers [80]. It has been described as a reduction in perinatal body fat in fetal cocaine exposed fetus, implying a restriction in nutrient fetal requirement. There are several possible mechanisms that may explain why this can occur in addition to maternal undernutrition: Cocaine can reduce amino acid uptake by the placenta and it can cause lack of nutrient supply through vasoconstriction of the uterine arteries [81]. Several recent observations suggest that prenatal cocaine exposure may impair the delivery of nutrients to the fetus. For example, cocaine abuse during pregnancy reduced placental sodium transport in mice, caused hyponatremia in human neonates, decreased uterine blood flow in rodents and sheep, reduced amino acid uptake in rat and human placentae [82], decreased the percentage of body fat content and the whole-body weights of rat fetuses and human infants [83], and altered bone composition in human and rat offspring [84].

MA can causes fetal growth restriction by several proposed mechanisms including placental vasoconstriction leading to restricted nutrient delivery to the fetus, fetal vasoconstriction and hypertension that decreases fetal oxyhemoglobin saturation [85].

Marijuana smoking, like tobacco smoking, can also increase carboxyhemoglobin levels which in turn may impair fetal oxygenation and, consequently, fetal growth [86]. Moreover, marijuana use tends to increase the heart rate and blood pressure which may reduce uteroplacental blood flow to the fetus [87]. Abnormalities in growth are biologically plausible, given the passage of cannabinoids across the placenta. There are some data suggesting that cannabis affects glucose and insulin regulation and, therefore, may affect the fetal growth trajectory [88].

Heroin is not considered to be grossly teratogenic, but it is highly lipophilic and readily crosses the placenta. Untreated heroin use is associated with IUGR, premature delivery, increased neonatal mortality and neonatal abstinence syndrome (NAS) [89].

Undernourished mother had a limited nutrient supply to the fetus. In utero malnutrition, if followed by low postnatal nutrition, can aggravate the effect of malnutrition, resulting in severe growth retardation and impaired neurological functions, such as IQ and language development [90].

Data conducted in experimental animals, demonstrated that deficiencies of certain nutrients, including folate, vitamin B12, Zn, Fe and Copper (Cu) during pregnancy can lead to suboptimal neurological outcomes and other abnormalities [91]. The metabolism of Fe, Zn and Cu are interrelated, so experimental animal models showed that if there is a maternal deficit of anyone it can lead to impairment in the metabolism of the other elements in the mother as well as the fetus [92]. This decreased average available for placental transport can result in fetal zinc and Cu deficiency that leads to IUGR and fetal dysmorphogenesis [43].

If maternal dietary calcium intake is low, fetal bone development and mineralization may be compromised if the same occurs for low intake of omega-3’s that may directly and adversely affect fetal neurological structures and cognitive function. Folate is a major requirement for brain and spinal cord development and riboflavin also plays a role in brain development. Vitamin B12 is critical to the process of DNA methylation and deficiencies in vitamin B12 can cause abnormalities in DNA methylation and neurodevelopmental deficits. The lack of these micronutrients in the fetus favor severe neurological disabilities. Choline deficiency during pregnancy is related to alcohol consumption and may cause motor deficiency and impairment of memory function in offspring [32]. The metabolism of Fe can also be impaired by a substance if combined with undernutrition status. Miller reported that ethanol consumption by rat dams perturbs the balance between Fe concentrations and Fe-regulatory proteins in brain regions of offspring [93]. Carter et al., showed that infants of binge drinking mothers had more risk to develop anemia due to iron deficiency, demonstrating disruption of the supply of Fe stores and postnatal impairment in iron metabolism [94]. Maternal vitamin A stores can also be depleted by alcohol consumption during pregnancy, impairing normal cell growth of the fetus. Retinol is essential for neuronal cell growth and transcription in the CNS [95]. In addition, nutrients such as folate, vitamin C (ascorbic acid), vitamin E (atocopherol), selenium, and zinc are antioxidants, so the low levels of these nutrients caused by alcohol and drugs of abuse can produce oxidative stress with consequent disruption of fetal development [17].

### 2.5. Nutrient Supplementation during Pregnancy

Nutrition is a protective factor against alcohol and drug of abuse teratogenity. When maternal nutritional status is altered, essential nutrients are unavailable for the fetus, and toxic harm of alcohol and drugs of abuse increases, this can result in developmental abnormalities, cognitive delays or FASD [96]. For these reasons, recent researches are focusing on nutritional intervention in order to reverse the detrimental effect of alcohol and drug of abuse on nutritional status of the mother with consequent impairment of fetal development. Probably micronutrient supplementation would in part reverse the toxic effect of alcohol and drugs of abuse on fetal neurological development, although it is difficult to establish the optimal range of micronutrients during pregnancy. Moreover, there are genetic polymorphisms that can vary nutritional requirement for every single woman. Choline and betaine (a metabolite of choline) are two of the most studied micronutrients for their importance in cognitive and memory function although there is not a known blood level of choline deficiency. A prospective clinical trial was conducted in Ukraine comparing a cohort of moderate/heavy drinking pregnant women and low /unexposed control pregnant women. Among the three groups treated by distinct multivitamin supplementation, the group who received choline supplementation experimented beneficial effects in fetus development and neurological outcome as basic learning and memory in the first year of life. Limitations of this study were the absence of recording dietary intake during pregnancy and the absence of a cohort receiving only choline supplementation [97].

Some experimental studies suggest treating women with minerals and antioxidants (like vitamin E or C) in order to substitute the nutritional deficits described in addicted mothers and to protect the fetus from the teratogenic effects of alcohol [98].

In animals, model supplementations with antioxidants to pregnant females reduced oxidative stress. Nutrients such as choline, vitamin E, bataine, folic acid, methionine, zinc can attenuate alcohol-induced changes to the epigenome and oxidative damage [99].

## 3. Limitations

One of the limitations of the present data is that this is a descriptive review and it is not a systematic review.

The studies found on the relationship of alcohol and nutrition are generally conducted in a specific population (South of Africa or Ukraine for example) that have lower economic status and this condition could be the majority cause of malnutrition.

For drugs of abuse it is difficult to establish in humans which is the mechanism of each specific substance that may affect mother nutrition, because addicted pregnant women often consume multiple drugs and have multiple co-infections like HIV, so the cause of mother undernutrition could be multifactorial.

Published studies about this subject in humans may have a potential bias because nutritional effects of alcohol or drugs of abuse might be caused not by the specific substance but to maternal low socioeconomic status.

## 4. Conclusions

To our knowledge, few studies report the effects of alcohol and drugs of abuse on maternal nutritional status. Although teratogenic effects of alcohol are well-known and despite public health efforts to reduce alcohol and drugs of abuse consumption during pregnancy, there is strong evidence that an important number of pregnant women continue to be addicted. Alcohol and drugs of abuse cause maternal and fetal malnutrition with consequent developmental fetal problems. It is crucial the maintain optimal nutrition during pregnancy in order to avoid detrimental effects on fetal neurostructural development. Recent researches are focusing on nutritional therapy in order to alleviate the effect of alcohol and drugs of abuse on nutritional status of the mother with consequent impairment on fetal development. Further studies are required to establish the role of nutritional intervention and the range of nutrition supplementation that could reverse the severity of the outcome of FASD, IUGR or fetal dysmorphogenesis.

Accordingly, this review tries to underline evidence of potential target nutrients for prenatal nutritional supplementation by focusing specifically on alcohol and drug metabolism effects on fetal development nutrient deficiency and their interactions with alcohol or drugs consumption. The identification of such nutrients can ameliorate the harmful effects of alcohol and drugs of abuse on fetus, preventing long-term disability. Appropriate screening for addiction in all women of childbearing age, preconception health promotion and referral to substance abuse programmes for those women identified to have an addicted disorder, could be done in all primary care settings. Programs of education and screening of women of childbearing age remain the most important ways to reduce addiction in pregnancy.

## Supplementary Materials

The following are available online at http://www.mdpi.com/2072-6643/10/8/1008/s1, Table S1: Studies about the effects of alcohol and drugs of abuse exposure on maternal nutritional status preconception and after conception period.
