# Supplementary material for: The Effects of Alcohol and Drugs of Abuse on Maternal Nutritional Profile during Pregnancy
Source: Nutrients. 2018 Aug 2;10(8):1008. doi: 10.3390/nu10081008 (PMC6116049; doi:10.3390/nu10081008)
Supplement: Supplementary file 1 [file nutrients-10-01008-s001.pdf]

Supplementary Materials: The Effects of Alcohol and Drugs of Abuse on Maternal Nutritional Profile During Pregnancy

Giorgia Sebastiani [1,*], Cristina Borrás-Novell [1], Miguel Alsina Casanova [1], Mireia Pascual Tutusaus [1], Silvia Ferrero Martínez [2], María Dolores Gómez Roig [2] and Oscar García-Algar [1]

Table 1. Studies about the effects of alcohol and drugs of abuse exposure on maternal nutritional status preconception and after conception period.

	Type of study	Nutritional status of mother	Key results	Reference
		**Alcohol Studies**		
Alcohol use and iron status in pregnant women	Case-control	Very heavy drinkers had:<br>-higher iron depletion<br>-not anemia	Drink pattern was not related to iron status except very heavy drinkers (> 8 drinks per day)	Streissguth et al. 198
Intake of micronutrients in non-pregnant alcohol abusers	Population research	Non-pregnant alcohol abusers had low intake vitamin A, B1,B2,B6, C, D, calcium, selenium, folic acid	Low intake of micronutrients in alcoholic non pregnant population	Manari et al. 2003
Maternal Risk factors for FAS before, during and after pregnancy	Population research case- control	FAS mother had less BMI before and during pregnancy than control mothers	FAS mothers had:<br>- More age<br>- Binge pattern of drinking or heavy drinking<br>- Longer drinking career before pregnancy<br>- Low BMI	May et al. 2004
Zinc and cupper levels of heavy drinking pregnant women	Case-control	Zn and Cu concentration were low in heavy drinking pregnant women	-Lower means values of minerals were observed in the alcohol exposed groups than in the controls	Keen et al. 2010
Nutrition profile in mothers of FASD children	Population research case- control	FASD mother had low intake of vitamin A, C, D, E, B2, calcium, omega-3, choline than control mothers	In FASD mothers<br>-Low BMI<br>- Inadequate intake of micronutrients	May et al. 2014
Alcohol exposure and maternal nutritional supplements	Case-control	Moderate to heavy drinking pregnant women had similar blood levels of choline than non-drinking mothers	Multivitamin supplementation was associated to better score of Bayley scale in 6- months babies	Coles et al. 2015
Maternal alcohol use and nutrition during pregnancy	Case-control	-Alcohol was related to higher intake of phosphorus, choline and vitamin B12<br>-Drinking frequency was associated with lower intake of vitamin C and occasional drink with more intake of vitamin D<br>- Alcohol was not related to anthropometric features	-All women gained less weight in pregnancy<br>- >85% of both group of pregnant women had insufficient intake for 10 of 22 key nutrients and >50% for additional 3 nutrients	Carter et al. 2016
		**Metamphetamine (MA) studies**		
Addiction in Pregnancy	Review	-Abuse have all been associated with poor maternal weight gain and nutritional status<br>-These substances are mixed, with powerful appetite suppressant properties.	MA use was associated to:<br>-Poor maternal nutritional status<br>-Fetal growth insufficiency	Keegan DO, et al. 2010

Effects of prenatal MA during gestation	Animal Study	-Sometimes little can be done to change established maternal behaviours in regard to nutrition<br>-Fetal growth insufficiency has been associated with MA use in pregnancy. It is unclear if this is related to a direct effect of the agent on the placenta or fetus or whether this represents a nutritional problem in patients who use MA<br>- Anorexia and blood pressure are increased by misuse of MA in mother<br>- MA abuse can decrease food intake and appetite<br>- Fetus and placenta weight decreased	- MA abuse increased anorexia<br>- Finally poor nutrition may affect pregnancy outcomes	Khoradmehr M.Sc, et al. 2015
Maternal alcohol and drugs abuse and nutrition during pregnancy	Case-control	Mother consuming MA showed:<br>-smaller biceps skinfold thickness<br>-lower BMI<br>-lower intake of vitamin C and carbohidrates		Carter et al. 2016
**Cocaine Studies**				
Maternal nutrition profile and daily cocaine administration	Animal Study	Cocaine administration was associated to:<br>-Less weight gain during pregnancy<br>-Weight recovery after parturition	- High risk of mother and fetus mortality at 60mg/kg/day cocaine.<br>- Less weight gain during pregnancy related to dosage.<br>- Pronounced lag in weight recovery after parturition	Wiggins et al. 1990
Serum illicit drug concentrations and maternal nutritional status	Population research	Addicted subjects had:<br>- lower serum folate and ferritine<br>- higher leukocyte levels	- Subjects whose serum values were above the ADAMHA/NIDA ranges for marijuana, PCP and cocaine had concentrations of folate and ferritin that were significantly lower than those of subjects with lower serum drugs levels.<br>- High maternal serum concentration of illicit drugs were accompanied by a significant increase in leukocyte count<br>- The level of maternal cocaine during the third trimester was inversely correlated with birth weight and head circumference	Knight et al. 1994
Maternal and fetal body composition related to cocaine	Animal studies	- Maternal weight gain and food consumption showed dose-dependent decreases	- Maternal weight gain and food consumption showed dose-dependent decreases<br>- Maternal water consumption, by contrast, was significantly increased<br>- Undernutrition was a sufficient cause of fetal weight reduction at dose of 50mg/kg/day	Church et al. 1995
**Cannabis studies**				
Effects of marihuana on the solution of anagrams, memory and appetite	Experimental study	Marihuana smokers consumed significantly more marshmallows	Subjects who smoked marihuana recalled significantly fewer items than the control subjects and there were significantly more erroneous recalls	Abel EL, 1971
Interactive effects of nutrition and cannabis upon rat perinatal	Clinical trial<br>Animal models	Female Wistar rats were exposed to cannabis smoke, placebo smoke, or no smoke while concurrently	-12 variables affected by the low-protein diet, 8 were significantly potentiated when under nutrition was combined with cannabis	Charlebois AT and Fried PA, 1980

development		consuming 1 of 3 diets differing in protein concentration (8%, 24%, 64%)	treatment<br>-these included a lengthened gestation period, an increase in occurrence of stillbirths and litter destruction, and decreased activity in the rat pups	
An investigation of prenatal cannabis exposure and minor physical anomalies in a low risk population	Case-control		None of the anomalies noted occurred more frequently among the offspring of cannabis users	O'Connell CM and Fried PA, 1984
Effects of maternal marijuana and cocaine use on fetal growth	Case-control		-Infants whose mothers had positive urine assays for marijuana, as compared with the infants whose mothers were negative according to both interviews and urine assays, had a 79-g decrease in birth weight and a 0.5-cm decrement in length<br>-Women who had positive assays for cocaine, as compared with nonusers, had infants with a 93-g decrease in birth weight (a 0.7-cm decrement in length and a 0.43-cm-smaller head circumference	Zuckerman B et al. 1989
Relationships of serum illicit drug concentrations during pregnancy to maternal nutritional status	Case-control study	Fasting blood samples, drawn during each trimester of pregnancy and at delivery, were screened for concentrations of cocaine, phencyclidine and marijuana Serum folate, vitamin B12, ferritin and ascorbic acid	-Subjects whose serum values were above the ADAMHA/NIDA ranges for marijuana, phencyclidine and cocaine had concentrations of folate and ferritin that were significantly less than those of subjects with lower serum drug levels	Knight EM et al. 1994
Characteristics of pregnant illicit drug users and associations between cannabis use and perinatal outcome in a population-based study	Population-based study	-Women who reported use of any illicit drug were less likely to have used folic acid in the periconception period<br>-Users were also more often underweight (BMI<18.5kg/m2) than women who did not report use of illicit drugs during pregnancy	-Cannabis users were more likely than nonusers to have excessive weight gain during pregnancy	Van Gelder MM et al. 2010
Marijuana Use and Maternal Experiences of Severe Nausea During Pregnancy in Hawaiʻi	Descriptive study	-6.0% reported using marijuana in the month before pregnancy<br>-2.6% reported using marijuana during pregnancy<br>-21.2% reported severe nausea during pregnancy	-Women who reported severe nausea during pregnancy were significantly more likely to report marijuana use during pregnancy (3.7% vs 2.3%)	Roberson E et al. 2014
		**Heroine studies**		
Burden and nutritional deficiencies in Opiate addicted	Review	-Unhealthy eating behaviors due to lack of nutritional knowledge, food preparation skills, and environments.<br>-During withdrawal from heroin, weight gain or loss occurs which is caused by major changes in food intake selection.<br>-Nutrition is related with conditions and diseases, such as diabetes which decreases sensitivity to dependence on morphine and vitamin D deficiency hat slows down morphine dependency as well as protein deprivation which generates preferential fat intake with low cocaine use.	-Opiate dependents have several deficiencies such as nutritional deficiencies and weight deficits -A good nutrition education and physical activity are quite effective for substance abusers to their withdrawal from opiates	Nabipour et al. 2014

		-Many opiate addicts have shown calcium and magnesium deficiencies		
Women of childbearing age and opioids	Report		-Outreach and educational resources targeting younger pregnant women and women living below the federal poverty level about the dangers of misusing prescription pain relievers may be especially beneficial	Smith K et al. 2017
